# Spatial versus spatio-temporal approaches for studying metacommunities: a multi-taxon analysis in Mediterranean and tropical temporary ponds

**DOI:** 10.1098/rspb.2023.2768

**Published:** 2024-04-03

**Authors:** Ángel Gálvez, Pedro R. Peres-Neto, Andreu Castillo-Escrivà, Fabián Bonilla, Antonio Camacho, Eduardo M. García-Roger, Sanda Iepure, Javier Miralles, Juan S. Monrós, Carla Olmo, Antonio Picazo, Carmen Rojo, Juan Rueda, Mahmood Sasa, Mati Segura, Xavier Armengol, Francesc Mesquita-Joanes

**Affiliations:** ^1^ Cavanilles Institute for Biodiversity and Evolutionary Biology, University of Valencia, Paterna, Valencia, Spain; ^2^ Department of Biology, Concordia University, Montreal, Canada; ^3^ Instituto Clodomiro Picado, Facultad de Microbiología, Universidad de Costa Rica, San José, 13, Costa Rica; ^4^ Department of Taxonomy and Ecology, University of Babes—Bolyia, Cluj Napoca, Romania; ^5^ Emil Racovitza Institute of Speleology, Cluj Napoca, Romania; ^6^ Centro GEMA—Genómica, Ecología & Medio Ambiente, Universidad Mayor, Santiago, Chile; ^7^ GRECO, Institute of Aquatic Ecology, University of Girona, Girona, Spain; ^8^ Museo de Zoología, Centro de Investigación en Biodiversidad y Ecología Tropical, Universidad de Costa Rica, San Jose, Costa Rica

**Keywords:** dispersal limitation, environmental selection, freshwater ecology, spatial variation, spatio-temporal variation, biogeography

## Abstract

Prior research on metacommunities has largely focused on snapshot surveys, often overlooking temporal dynamics. In this study, our aim was to compare the insights obtained from metacommunity analyses based on a spatial approach repeated over time, with a spatio-temporal approach that consolidates all data into a single model. We empirically assessed the influence of temporal variation in the environment and spatial connectivity on the structure of metacommunities in tropical and Mediterranean temporary ponds. Employing a standardized methodology across both regions, we surveyed multiple freshwater taxa in three time periods within the same hydrological year from multiple temporary ponds in each region. To evaluate how environmental, spatial and temporal influences vary between the two approaches, we used nonlinear variation partitioning analyses based on generalized additive models. Overall, this study underscores the importance of adopting spatio-temporal analytics to better understand the processes shaping metacommunities. While the spatial approach suggested that environmental factors had a greater influence, our spatio-temporal analysis revealed that spatial connectivity was the primary driver influencing metacommunity structure in both regions. Temporal effects were equally important as environmental effects, suggesting a significant role of ecological succession in metacommunity structure.

## 1. Introduction

The metacommunity concept has changed how ecologists study species distributions by incorporating multiple processes such as environmental selection, dispersal and ecological drift [1–3]. While it is recognized that metacommunity structure changes over time [4,5], most research has predominantly focused on snapshots of groups of local communities (sites) at a single time period [6,7]; therefore, analysing their temporal dynamics should offer a more thorough understanding of underlying processes [8].

Some metacommunity studies have examined how spatial variation in species composition changes across different time periods, a design we refer to as a ‘spatial approach’ [5,9,10]. These ‘snapshot’ studies have shown that shifts in spatial connectivity and environmental heterogeneity can influence the structure of local communities. An increase in connectivity often leads to community homogenization, which may result in reduced beta diversity and increased neutral mass effects. As a result, species distributions can be more accurately predicted from environmental variation (heterogeneity) at intermediate levels of connectivity, but predictions become less reliable at both high and low levels of connectivity [11]. Conversely, environmental heterogeneity can increase spatial beta diversity and generate strong associations between species distributions and environmental conditions. However, these repeated snapshot analyses predominantly focus on spatial variation, overlooking the complex interplay between temporal and spatial variations within metacommunities.

Other studies have investigated metacommunity variation across both spatial and temporal dimensions, incorporating time itself as an explanatory variable, a design we refer to as a ‘spatio-temporal approach’ [12–16]. This approach facilitates estimating the importance of environmental, spatial and temporal processes, such as succession and historical events, in structuring metacommunities, all within a single analytical model that considers the three axes of variation explicitly. Spatio-temporal studies have generally found that environmental (e.g. species sorting) and spatial processes (e.g. dispersal) exert a greater influence than temporal processes in shaping metacommunity variation. However, significant temporal effects have been identified in some cases [12–14] (but see [15]). Additionally, temporal effects can be influenced by environmental factors that themselves vary over time [16], whether seasonally, annually or even stochastically. However, it is worth noting that spatio-temporal analyses are much less common compared with snapshot spatial studies, and the development of suitable statistical methods for them is relatively recent (e.g. computing eigenfunctions for spatio-temporal variables) [17,18].

Temporary ponds, known for their temporal variability, serve as valuable systems for studying temporal processes in metacommunities, such as dormancy, priority effects and seasonal dynamics associated with the hydroregime [19–23]. Climate seasonality can have substantial temporal or spatio-temporal impacts on metacommunity variation, influencing environmental and spatial connectivity. While ponds undergo regular fluctuations in water levels, variations in precipitation, temperature and species adaptability to these conditions could lead to different processes structuring metacommunities across regions. Some studies have shown that temperate regions exhibit greater environmental heterogeneity over time, including factors such as temperature seasonality, in comparison to tropical regions [24]. This greater heterogeneity has been suggested as the primary reason for the more pronounced environmental effects on metacommunities in temperate regions compared to tropical ones [25]. By contrast, tropical freshwater ecosystems may experience drastic changes in spatial connectivity due to seasonal heavy rains. Extreme rainfall events can enhance pond interconnectivity through floods, thereby promoting metacommunity homogenization, and resulting in reduced environmental and spatial effects [5,26,27].

Previous research has explored the influence of environmental and spatial factors on aquatic metacommunities across various bioclimatic regions; however, studies employing standardized sampling designs across these regions are particularly rare [28,29]. Some studies (e.g. [25]) underscore that environmental factors have a greater influence on temperate metacommunities, while spatial factors play a dominant role in shaping tropical metacommunities [26,27]. These pronounced spatial effects in the tropics, often attributed to dispersal limitation, can be linked to multiple factors: the stronger orographic barriers in the tropics, which affect organisms adapted to low temperature seasonality, known as the Janzen effect [28]; patchy species distributions less influenced by local environmental conditions [30]; and increased pond connectivity during rainy seasons [14]. However, it is important to acknowledge that the processes influencing metacommunities can shift over time [29,31], suggesting the need for further exploration of these snapshot studies' findings through the lens of temporal dynamics.

Our primary goal here was to evaluate insights obtained from metacommunity analyses based on a static spatial approach repeated over time, which compares the importance of environmental and spatial variation in each time period separately, with a more robust spatio-temporal approach that consolidates all data into a single unified model. To do so, we designed a study to assess the influence of temporal variation in the environment and spatial connectivity on the structure of metacommunities in tropical and Mediterranean temporary ponds. Employing a standardized sampling methodology across both regions, we collected samples of multiple freshwater taxa in three time periods within the same hydrological year from multiple temporary ponds in each region. To evaluate how estimates of the influence of environmental, spatial and temporal variation on community structure vary between the two approaches, we developed a nonlinear framework for variation partitioning based on generalized additive models (GAMs). Under the static spatial approach repeated over time, we anticipated differences in the relative roles of environmental and spatial effects across sampling seasons. Specifically, we expected that environmental factors would play a more dominant role in the Mediterranean metacommunity and spatial factors would be more influential in the tropical metacommunity, regardless of the sampling period. In the spatio-temporal approach, we predicted that while temporal variation would significantly influence metacommunity structure, its influence would be less than that of environmental and spatial factors. Furthermore, we expected temporally structured environmental variation to be more important in the Mediterranean metacommunity, especially due to seasonal changes in temperature in this biogeographic region.

## 2. Material and methods

### (a) Study area and sampling method

**Figure 1.**
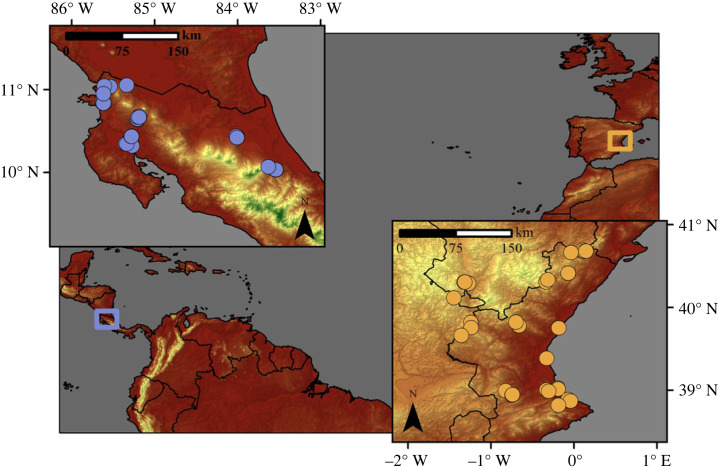
Map of the study area showing the locations of the ponds sampled in each metacommunity: tropical (Costa Rica) and Mediterranean (eastern Spain).

In this study, we surveyed 30 tropical temporary ponds in Costa Rica and 32 Mediterranean temporary ponds in Eastern Spain (figure 1). We focused on shallow waterbodies (less than 2 m of depth) with fresh or oligohaline (less than 7000 µS cm^–1^) water, including small, naturalized farmland ponds, inland shallow lakes and coastal wetlands. These ponds were selected for their varying hydroperiods, with most undergoing an annual dry period of few months (21 and 23 ponds in the tropical and in the Mediterranean region, respectively). However, a few have semipermanent hydroperiods that last extended periods (a few years; 9 ponds in each region). The tropical ponds were situated in a region with warm temperatures throughout the year (24.9 ± 1.3°C) and high, albeit seasonally variable, precipitation (2486 ± 934.7 mm). By contrast, the Mediterranean ponds were located in a region with cooler average annual temperatures (12.9 ± 3.0°C) and lower, yet seasonally variable, annual precipitation (537 ± 68.3 mm) [32].

Each pond was surveyed three times during the flooding period within the same hydrological year, occurring in early (approximately two weeks after the ponds were filled, in May 2017 for Costa Rica and January 2018 for Spain), mid (October 2017 and April 2018, respectively) and late (just before the ponds with shorter hydroperiods dried up; January 2018 for Costa Rica and June 2018 for Spain) stages of the hydroperiod. These ponds were spread across a similar spatial range in both regions, covering areas of 10 000 km^2^ and 13 000 km^2^ in Costa Rica and Spain, respectively, and spanning in altitude from sea level to 1500 m above sea level.

We collected presence–absence data for seven major groups of aquatic organisms, representing the primary taxa found in these ponds: bacteria and archaea from the water column, phytoplankton, rotifers, microcrustaceans (including branchiopods, copepods and ostracods), benthic macroinvertebrates and amphibians. The community composition of bacteria and archaea was determined using the presence of different zero-radius operational taxonomic units (ZOTUs) via next-generation sequencing, as detailed in [33]. Whenever possible, species-level identification was performed for the other groups. Further information about the sampling methods and sample processing is available in [14].

We assessed a range of limnological (e.g. dissolved oxygen concentration, electrical conductivity, pH, nutrient concentration, etc.), hydrogeomorphological (e.g. depth, diameter, sediment composition), biotic (e.g. fish or livestock presence), climatic and landscape (e.g. land uses around the pond) variables for environmental characterization, as detailed in [14] and [34]. For subsequent analyses, we processed these environmental variables, either logarithmically or through the arcsine of the square root transformation, depending on their frequency distribution. This was done to minimize the leverage and skewness caused by extreme values in these predictor variables [35]. The pond characterization and biological data are available at Figshare [36].

### (b) Statistical analyses

The metacommunity structure of each of the seven main groups of aquatic organisms (bacteria, archaea, phytoplankton, rotifers, microcrustaceans, macroinvertebrates and amphibians) was analysed using two approaches: spatial and spatio-temporal (see below). We enhanced the traditional method of variation partitioning [37], a multivariate regression approach that considers multiple predictor matrices for multiple responses (species) in metacommunity analyses, through two modifications. The first modification was intended to address the issue that in the standard variation partitioning framework, environmental predictors are treated linearly, while spatial and temporal predictors are handled in a nonlinear fashion. By employing nonlinear GAMs instead of multiple linear regressions, we improved our ability to estimate the relative importance of environmental selection (species sorting) versus spatial and temporal processes in structuring the two studied metacommunities across the different taxonomic groups [14,38]. The second modification was to improve the representation of the response variable, i.e. the species distribution (community) matrix. Here, we used the predicted values from a generalized linear latent variable model (GLLVM) using a binomial distribution with no predictor variables [39]) rather than using the original community data as response matrix. These axes result in a more robust method for community analyses because the latent (GLLVM) axes better capture patterns of covariation among species, thereby reducing independent variation not shared by species in the analyses. The standard variation partitioning approach models the covariation and independent variation across all species. The number of latent axes to be used as a response matrix for each any given taxon was determined by the combination leading to the lowest AIC [39]. Similar to standard variation partitioning, independent GAM models were generated for each latent axis separately, and a canonical (global) adjusted *R*^2^ was calculated as the average across models [37].

Initially, using a purely spatial approach, we analysed each of the three sampling seasons as a separate snapshot survey to evaluate how the importance of environmental and spatial factors (predictors in variation partitioning) varied over the hydroperiod. Subsequently, we reanalysed the metacommunity using a spatio-temporal approach, partitioning the variation observed across all three sampling seasons together, using predictor matrices that included environmental, spatial and temporal predictors [40]. These analyses were performed separately for each region and each group of organisms.

When using GAMs for the spatial approach, we were faced with the limitation of including a maximum of three environmental predictors (each with nine splines per variable) due to the sample size our of study (i.e. about 30 ponds per region and period). To address this issue, we summarized the environmental variation using their first three principal components (PCs) and incorporated them as environmental predictors in the GAM models. As spatial predictors, we used splines of longitude and latitude to model complex nonlinear spatial patterns. Additionally, to further reduce the number of environmental and spatial predictors) in the models, we used a forward selection method for GAMs with a double-stopping criterion [41]. This process involved two steps: initially, we selected only those predictors that were statistically significant (*p*-value < 0.05). Subsequently, we verified that the adjusted *R*^2^ of the selected predictors (i.e. their splines) was lower than the adjusted *R*^2^ of the model incorporating all splines. We repeated this procedure separately for the environmental (three PCs) and spatial variables. In rare instances where no predictor was selected because the double-stopping criterion was not met, we included at least one environmental or spatial predictor, provided it was statistically significant. Due to the limitation of three predictor variables considering the number of ponds in the spatial approach, we manually reduced the number of variables (either spatial or environmental) if the model selection procedure initially chose more than three. We repeated this process separately for each sampling season and for each group of organisms within each region.

Based on the adjusted *R*^2^ values obtained from the GAMs, we estimated the proportion of total metacommunity variation explained by environmental (E) and spatial (S) components [37] separately for each of the three time periods (i.e. our spatial approach), as well as their pure fractions. These included the pure environmental effects independent of space (E | S), interpreted as the component related to environmental selection, and the pure spatial effects independent of the environment (S | E), commonly interpreted as the result of processes limiting dispersal. Finally, we calculated the common fraction of variation explained by both environmental and spatial components (E ∩ S), interpreted here as variation due to the spatial structure of the environment [42]. The unexplained proportion of variation (residuals) represents sources of variation among species not accounted by the measured environmental or spatial variables. To mitigate potential spurious correlations between species distributions and spatially structured environments (as discussed in [43]), we implemented the correction method for the environmental component proposed in [44]. To ensure comparability of results among taxa and regions, we converted the fractions of explained variation into relative proportions by dividing each unique and common fraction by the total variation explained by their respective models (E + S).

For the spatio-temporal approach, we consolidated all the data into a single unified model which incorporates a temporal dimension into the variation partitioning. This temporal dimension is represented by the number of days since the first sampling of each pond (i.e. in initial sampling season). This unified model allowed us to increase the number of predictors by combining data from all ponds over time within a given region across three time periods. As with the previous approach, we condensed the number of environmental variables by using the first three PC axes of the environmental predictors. As in the spatial approach, we used splines (i.e. GAMs) of these three PCs and splines of latitude and longitude as environmental and spatial predictors, respectively. With the inclusion of a temporal component (T), variation partitioning estimated the total explained variation (E + S + T) and unique fractions explained by each component: pure environmental effects (E | (S + T)), pure spatial effects (S | (E + T)) and purely temporal effects (T | (E + S)). Common fractions are interpreted here as spatially structured environment ((E ∩ S) | T), temporally structured environment ((E ∩ T) | S), spatio-temporal overlap ((S ∩ T) | E) and spatio-temporal structured environment (E ∩ S ∩ T). As in the previous approach, to control for spurious correlations between species distributions and spatially and/or temporally structured environments, we applied the correction method for the environmental component developed by [44] (see also [18] for applying this method using spatio-temporal variation). Again, to ensure comparability of results among taxa and regions, we converted the fractions of explained variation into relative proportions by dividing each pure or overlapped fraction by the total explained variation (E + S + T). To assess differences in the proportion of variation explained by pure spatial effects and pure environmental effects between the spatial and spatio-temporal approaches, we used paired Wilcoxon tests. These tests were applied to all taxonomic groups, separately for both the tropical and Mediterranean metacommunities.

Lastly, we compared the differences in environmental heterogeneity between regions, examining each sampling period individually and then all three periods collectively, using tests of homogeneity of multivariate dispersion (PERMDISP) [45]. For this analysis, we were able to use all measured environmental variables, performing separate analyses for climatic variables (which are constant over the hydroperiod and expected to be more spatialized) and limnological variables (which are likely to exhibit greater local variability over time). This allowed us to generate measures of total environmental, climatic and limnological heterogeneity. All analyses were performed with R v4.0.2 [46] and R packages vegan [47], ade4 [48], adespatial [49], gllvm [50] and mgcv [51]. Codes for variation partitioning analyses can be found at Zenodo for the spatial approach [52] and for the spatio-temporal approach [53].

## 3. Results

**Figure 2.**
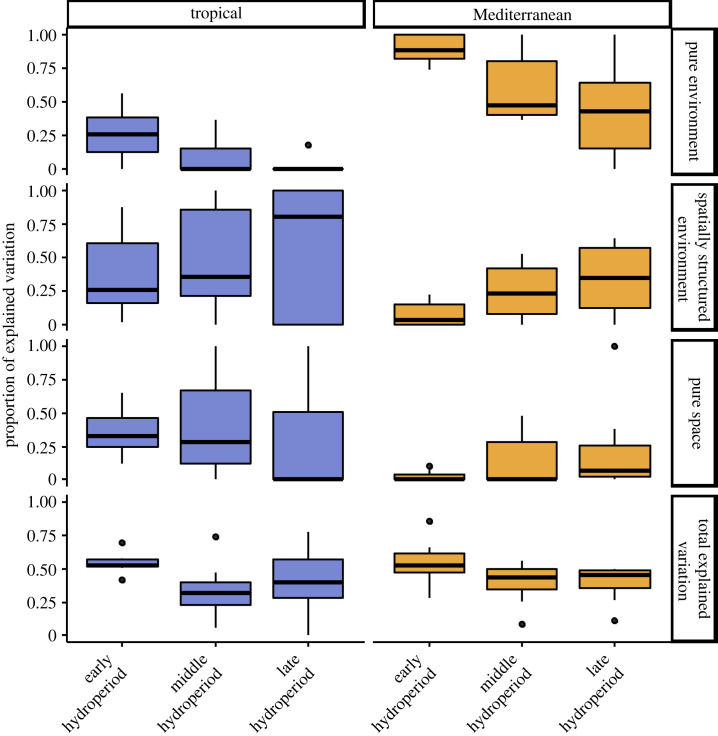
Results from the variation partitioning analyses for each biogeographic region (metacommunity) and sampling period, featuring boxplots depicting the relative contributions of pure environment (E | S) / (E + S), spatially structured environment (E ∩ S) / (E + S), pure space (S | E) / (E + S) and total explained variation (E + S). For values for each group of organisms, refer to electronic supplementary material, table S1.

**Figure 3.**
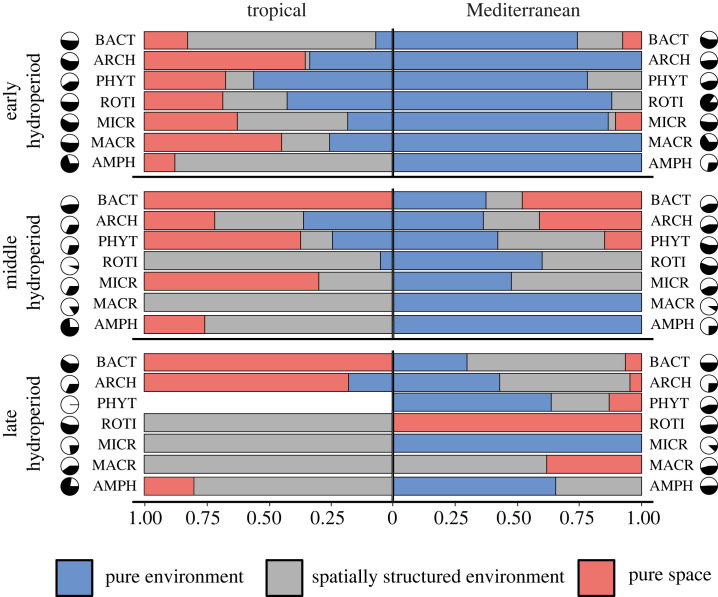
Results from the variation partitioning analyses for each taxon in each biogeographic region and sampling period (spatial approach). The results show the relative contributions of pure environment (E | S) / (E + S), spatially structured environment (E ∩ S) / (E + S) and pure space (S | E) / (E + S), each represented with a different colour. In the pie charts, black segments indicate the total proportions of explained variation, while the white segments represent the residual (unexplained) variation for each taxon: bacteria (BACT), archaea (ARCH), phytoplankton (PHYT), rotifers (ROTI), microcrustaceans (MICR), macroinvertebrates (MACR) and amphibians (AMPH).

The spatial approach suggests that the importance of environmental selection (pure environmental effects to the total variation) in both regions decreased over the hydroperiod, while spatial effects or their common variation exhibited a slight increase (figure 2). The Mediterranean metacommunity had a higher fraction of variation attributable to pure environmental effects. Conversely, pure spatial and spatially structured environmental effects were more pronounced in the tropical ponds (figure 2). These patterns generally remained consistent across all sampling periods, although not every taxon followed a similar trend, with some showing high variability over time (figure 3; electronic supplementary material, table S1). There were no statistically significant differences in the total environmental heterogeneity between regions at any sampling period, nor between sampling periods within the same region (PERMDISP tests; electronic supplementary material, table S2). However, climatic heterogeneity was greater in the tropical ponds, whereas limnological heterogeneity was more pronounced in the Mediterranean metacommunity (in this case only significantly during the early and late hydroperiods).

**Figure 4.**
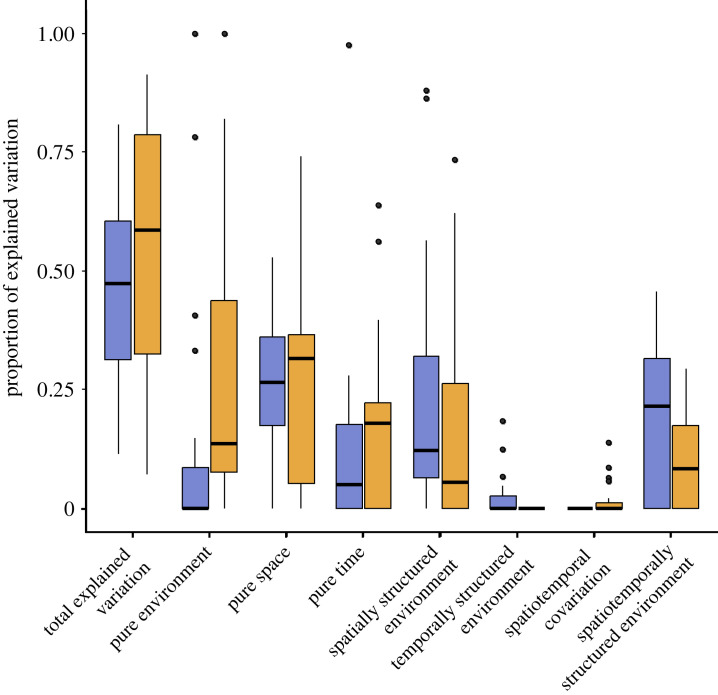
Boxplot results from the variation partitioning analyses for each biogeographic region (metacommunity), including the temporal component (spatio-temporal approach), are presented. These results show the total explained variation (E + S + T) and relative contribution of pure environment (E | (S + T)) / (E + S + T), pure space (S | (E + T)) / (E + S + T), pure time (T | (E + S)) / (E + S + T) and spatially structured environmental effects ((E ∩ S) | T) / (E + S + T), temporally structured environmental effects ((E ∩ T) | S) / (E + S + T), variation explained due to spatio-temporal covariation ((S ∩ T) | E) / (E + S + T) and the spatio-temporally structured environmental effects (E ∩ S ∩ T) / (E + S + T). Each region is represented by a different colour: tropical in blue and Mediterranean in orange. For detailed values for each group of organisms, refer to electronic supplementary material, table S3.

In the spatio-temporal approach, which involved analysing all three sampling periods collectively by considering a temporal variable as a third component in the variation partitioning analyses, we observed that the total proportion of explained variation was higher than when analysing each sampling period separately, as in the spatial approach previously described. Moreover, pure temporal effects played an important role in shaping metacommunity structure and were in par with pure environmental effects in both metacommunities (figure 4). The most influential factor in metacommunity organization, however, was pure spatial variation, showing no differences between the tropical and Mediterranean systems. Spatially structured environmental variation was also important in both cases. The effects of the spatio-temporal structure of the environment were substantial, especially in the tropics. The Mediterranean metacommunity exhibited greater structuring by pure temporal and environmental effects compared to the tropical metacommunity. Finally, the common variation between space and time, and between environment and time, was negligible in both regions.

**Figure 5.**
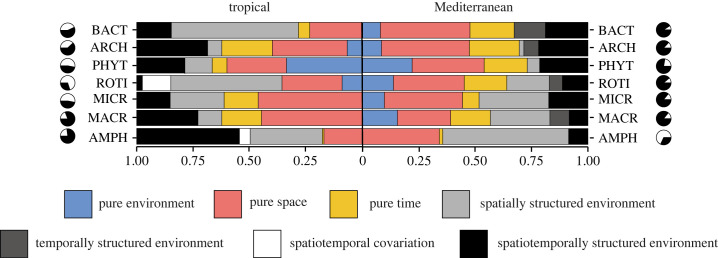
Results from the variation partitioning analysis, consolidating all sampling periods for each taxonomic group in tropical and Mediterranean metacommunities. It depicts the relative contribution of pure environment (E | (S + T)) / (E + S + T), pure space (S | (E + T)) / (E + S + T), pure time (T | (E + S)) / (E + S + T), spatially structured environmental effects ((E ∩ S) | T) / (E + S + T), temporally structured environmental effects ((E ∩ T) | S) / (E + S + T), variation explained due to spatio-temporal covariation ((S ∩ T) | E) / (E + S + T) and the spatio-temporally structured environmental effects (E ∩ S ∩ T) / (E + S + T). Each type of contribution is represented by a different colour. In the pie charts, black segments show the total proportions of explained variation, while white segments indicate the residual variation for each taxon: bacteria (BACT), archaea (ARCH), phytoplankton (PHYT), rotifers (ROTI), microcrustaceans (MICR), macroinvertebrates (MACR) and amphibians (AMPH).

**Figure 6.**
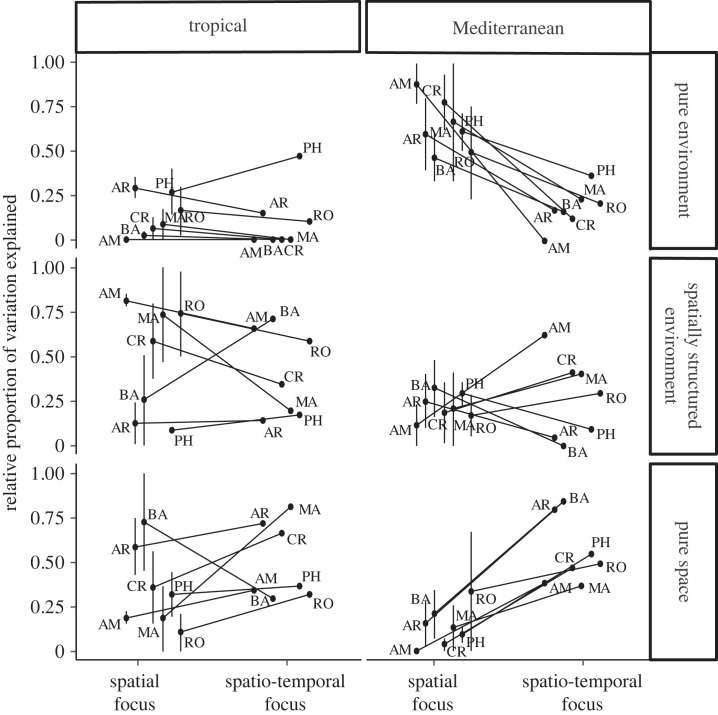
Comparison of results obtained for purely environmental (E | S) / (E + S), spatially structured environmental (E ∩ S) / (E + S) and spatial effects (S | E) / (E + S) between the spatial and the spatio-temporal approaches. The spatial approach results are shown as the average between the three sampling periods ± s.e. (standard error), while the spatio-temporal approach results have been recalculated after excluding the temporal effects. This allows contrasting the two approaches in their environmental and spatial contributions for each taxon: bacteria (BA), archaea (AR), phytoplankton (PH), rotifers (RO), microcrustaceans (CR), macroinvertebrates (MA) and amphibians (AM).

The proportion of variation explained by the different sets of predictors in the spatio-temporal approach varied considerably among taxa and regions, deviating from the patterns observed in analyses that focused solely on separate spatial snapshots (i.e. spatial approach; figure 5 versus figure 3; electronic supplementary material, table S3). When comparing the environmental and spatial fractions of the variation explained by both methods, we noted an increase in the proportion of pure spatial effects in the spatio-temporal approach, while pure environmental effects decreased. These differences were more pronounced in the Mediterranean metacommunity and became even more apparent when the temporal proportion was excluded (figure 6). Wilcoxon test results indicated no significant differences between the spatial and spatio-temporal approaches in the tropical metacommunity (*p* > 0.05 for the comparison of pure spatial effects, pure environmental effects and their overlap). However, for the Mediterranean metacommunity, significant differences were found in the pure spatial and pure environmental effects (*V* = 28, *V* = 0, respectively, *p* = 0.016 in both cases), but not in their overlap.

## 4. Discussion

A central goal of this study was to improve our understanding about the roles of environmental, spatial and temporal factors in shaping the metacommunity structure of a diverse range of taxa in two distinct biogeographical regions. Through the exclusive use of a spatial approach, our findings revealed a diminishing importance of pure environmental effects through time. Two primary factors, environmental heterogeneity and connectivity, are considered as critical in shaping metacommunity structure [3]. While previous studies have posited that increased environmental heterogeneity leads to stronger environmental selection [54,55], our results do not entirely align with this observation. The decrease in pure environmental effects in these metacommunities throughout the hydroperiod did not align with a reduction in overall environmental heterogeneity. However, we observed a growing influence of spatially structured environment concurrent with the decline in pure environmental effects. This suggests a possible shift from more localized environmental effects, which are independent of spatial patters (e.g. pond area and depth, type of substrate, organic matter, egg bank status, initial limnological conditions) to broader or aggregated environmental factors such as precipitation or temperature. These broader effects might be intertwined with spatial effects, potentially linked to a change in dispersal and colonization dynamics, constrained environmentally to aggregated pond clusters.

An increase in connectivity can lead to reduced environmental effects through metacommunity homogenization [11]. Connectivity shifts, particularly among spatially proximate ponds, may occur in the tropical metacommunity during the rainy season, as observed in other tropical studies [28]. However, metacommunity homogenization resulting from increased connectivity is typically associated with environmental homogenization [26], which was not observed in our studied metacommunities. In addition to hydrology, factors such as active dispersal in flying insects [56] or passive dispersal mediated by mammals and birds [57–59] may increase as the hydroperiod advances. However, these dispersal events are expected to lead to a reduction in beta diversity [60], but such a decline was not observed in previous analyses of these same metacommunities [61]. In Mediterranean ponds, metacommunity homogenization due to increased connectivity is less likely, given their physical isolation throughout the hydrological year.

The observed decrease in pure environmental effects over the hydroperiod may also be attributed to the growing influence of unmeasured environmental variables, particularly those influencing biotic interactions [62]. Strong shifts in metacommunity structure can be driven by community succession, leading to the development of new biotic interactions [63]. As a result, biotic interactions may become increasingly important over the hydroperiod as communities become more complex [64]. Ignoring these interactions could result in an overestimation of either the residual variation or the pure spatial effects [65,66].

Our results show that the Mediterranean metacommunity exhibits more pronounced pure environmental effects. This observation aligns with our initial findings from the first sampling season [14]. Importantly, we validate this pattern through both a spatial analysis over the entire hydrological cycle (i.e. three sampling periods) and a spatio-temporal approach. The few previous studies comparing metacommunity organization across different biogeographical regions have also found stronger environmental selection in temperate areas [25,67], possibly due to greater environmental heterogeneity, particularly in temperature regimes [30]. However, our study reveals no notable differences in overall environmental heterogeneity between the two metacommunities. The Mediterranean region exhibits higher limnological heterogeneity due to local factors, contributing to its greater pure environmental effects compared to the tropical ponds. Conversely, the tropical region displays greater climatic heterogeneity driven by regional factors (e.g. climate), leading to more pronounced spatially structured environmental effects.

Our research indicates that spatial factors exert a more pronounced influence on the tropical metacommunity than on the Mediterranean one, a trend consistent across sampling seasons. This finding aligns with our earlier study [14] and is supported by other works [25,67]. The stronger orographic barriers in the tropics (such as the Continental Divide in Costa Rica and the Janzen effect [28]), may limit dispersal, resulting in more limited species distributions compared to temperate regions [68]. Additionally, the tropics feature more distinct spatial patterns in environmental variables, like climate components, [69], and experience shifts in regional connectivity during the rainy season [28].

The spatio-temporal approach determined that pure temporal effects (dynamics) are as important as pure environmental effects in shaping metacommunity structure. This suggests that changes in metacommunity composition over time (e.g. succession, phenology), occur independently of temporal variation in the environment. Additionally, we observed that these pure temporal effects were slightly more pronounced in the Mediterranean metacommunity than in the tropical one. This suggests that the presence of certain species in the Mediterranean metacommunity is more influenced by the timing of the hydroperiod than in the tropical metacommunity. The high unpredictability of Mediterranean ponds [70] might lead to more varied ecological succession in these ecosystems, as opposed to the more stable tropical ecosystems.

In our spatio-temporal approach, pure spatial effects emerged as the most important component in both tropical and Mediterranean metacommunities. Therefore, it appears that pure spatial processes, primarily associated with dispersal limitation as suggested in [42] (and possibly encompassing other unmeasured abiotic and biotic factors that are spatially structured), are probably the main drivers of metacommunity structure in temporary ponds, as proposed by [71]. This observation contradicts our findings from a solely spatial approach, where pure spatial effects were typically less pronounced than pure environmental effects within the Mediterranean metacommunity. Consequently, the spatio-temporal approach might be more effective in identifying recurring processes that influence species distributions across locations over time, and it could better reveal dispersal limitations associated with species that have restricted distributions. Incorporating temporal effects reduced the relative influence of the environment (in favour of spatial processes) in the spatio-temporal approach. This shift highlights the importance of species persistence at specific sites, which may be linked to priority effects. The overlapping fractions of explained variation between environmental, spatial and temporal factors, interpreted as environmental effects structured by space–time, constitute most of the environmental influences on the organization of temporary pond metacommunities [72]. In the Mediterranean metacommunity, pure environmental effects are more pronounced, aligning with expectations of higher environmental variability in temperate regions, particularly regarding temperature variations [28,30,73,74]. However, the spatially and temporally structured environmental variation was slightly more prominent in the tropical metacommunity. This suggests that seasonality plays a significant role in the tropical ponds, underscoring the importance of spatially variable environmental factors [68].

When we incorporated the temporal component, namely the spatio-temporal approach, we observed a lower proportion of residual (unexplained) variation compared to using each sample period separately (i.e. the spatial approach). This suggests that incorporating time enhances the detection of community composition patterns and provides a more comprehensive explanation of the processes shaping these metacommunities [8]. However, it is worth noting that differences in total explained variation might stem from distinct sample size between both approaches.

While some studies have detected temporal effects through extensive temporal series or palaeoecological data [13], our study demonstrates that, within a relatively short time frame, significant portions of variation—exceeding those observed in other studies—are attributable to pure temporal effects. This could be due to the pronounced temporal dynamics characteristic of these temporary ponds. Altogether, including temporal variation in metacommunity studies seem critical for understanding the variability of structuring processes over time and for addressing the combined roles of environment, space and time in shaping the structure of metacommunities [8].

## 5. Conclusion

Our study of temporary ponds supports the long-standing view that environmental selection has a more pronounced role in temperate metacommunities than in tropical ones, where spatial effects such as dispersal limitation, are more dominant. However, we observed a decline in the influence of environmental selection throughout the hydroperiod, accompanied by an increase in the effects of spatially structured environmental variation. This suggests that over the hydroperiod, local abiotic factors may decrease in relevance, giving way to unmeasured environmentally mediated processes such as biotic interactions or dispersal.

Our study demonstrated that a spatio-temporal approach, which aggregates all sampling periods into a single model that includes a temporal predictor, enhances our ability to reveal the processes structuring a metacommunity. Moreover, by examining two distinct biogeographical regions, we could contrast how they differ in the environmental, spatial and temporal structuring of their respective metacommunities. It resulted in a higher proportion of explained variation compared to the spatial approach. Additionally, variation partitioning demonstrated that temporal effects are not merely secondary processes but are as important as environmental or spatial effects in influencing these metacommunities. Past studies that employed a spatial approach, without aggregating all sampled data into a single model, may benefit from re-evaluating their results using the single-model spatio-temporal approach implemented in our study.

## Data Availability

Raw data can be found at Figshare [36]. Codes for variation partitioning analyses can be found at Zenodo [52,53]. Supplementary material is available online [75].
